# 3D-Printing of Microfibrous Porous Scaffolds Based on Hybrid Approaches for Bone Tissue Engineering

**DOI:** 10.3390/polym10070807

**Published:** 2018-07-23

**Authors:** Ranjith Kumar Kankala, Xiao-Ming Xu, Chen-Guang Liu, Ai-Zheng Chen, Shi-Bin Wang

**Affiliations:** 1 Institute of Biomaterials and Tissue Egineering, Huaqiao University, Xiamen 361021, China; ranjithkankala@hqu.edu.cn (R.K.K.); 17013087034@hqu.edu.cn (X.-M.X.); 1611315017@hqu.edu.cn (C.-G.L.); 2 Fujian Provincial Key Laboratory of Biochemical Technology (Huaqiao University), Xiamen 361021, China

**Keywords:** 3D-printing, gelatin, nano-hydroxyapatite, poly(lactide-*co*-glycolide), osteoblast differentiation, polymeric scaffolds

## Abstract

In recent times, tremendous progress has been evidenced by the advancements in various methods of generating three-dimensional (3D) porous scaffolds. However, the applicability of most of the traditional approaches intended for generating these biomimetic scaffolds is limited due to poor resolution and strict requirements in choosing materials. In this work, we fabricated 3D porous scaffolds based on the composite inks of gelatin (Gel), nano-hydroxyapatite (n-HA), and poly(lactide-*co*-glycolide) (PLGA) using an innovative hybrid strategy based on 3D printing and freeze-drying technologies for bone tissue engineering. Initially, the PLGA scaffolds were printed using the 3D printing method, and they were then coated with the Gel/n-HA complex, yielding the Gel/n-HA/PLGA scaffolds. These Gel/n-HA/PLGA scaffolds with exceptional biodegradation, mechanical properties, and biocompatibility have enabled osteoblasts (MC3T3-E1) for their convenient adhesion as a layer and have efficiently promoted their growth, as well as differentiation. We further demonstrated the bone growth by measuring the particular biomarkers that act as key players in the ossification process (i.e., alkaline phosphatase (ALP), osteocalcin (OC), and collagen type-I (COL-I)) and the total proteins of the MC3T3-E1 cells. We anticipate that the convenient generation of highly porous 3D scaffolds based on Gel/n-HA/PLGA fabricated through an innovative combinatorial approach of 3D printing technology and freeze-drying methods may undoubtedly find widespread applications in regenerative medicine.

## 1. Introduction

The reconstruction of bone with suitable scaffolds that mimic the native bone tissues has garnered increasing interest for treating patients with bone defects. More often, the repair of bone defects and the reconstruction of bone tissue happen to be favorable through the degradation and absorption of the scaffolds [1,2]. However, the applicability of these scaffolds is often limited due to inadequate autogenous bone, poor biodegradability, and the risk of immune rejection in most instances. Therefore, the generation of suitable engineered three-dimensional (3D) porous scaffolds with a specific structure that meets the needs of patients with different bone defects is a critical need. In addition, these scaffolds would undoubtedly gain great significance in the clinical practice. Along this line, synthetic biomaterials have been used in the past decade as bone graft substitutes due to their excellent biomechanical properties, significant functional attributes, and efficient augmentation of bone growth and differentiation [3]. In this vein, various advancements have been made in the generation of these scaffolds by loading growth factors, drugs, stem cells, and genes, which can efficiently promote osteogenic differentiation and angiogenesis [4,5,6,7]. To improve the physicochemical attributes, as well as the mechanical properties, of the scaffolds and accelerate the rate of osteogenesis, numerous materials have been incorporated in a variety of engineered scaffolds that have been constructed from different materials based various synthetic polymeric materials [8,9,10].

Several approaches have so far been developed to prepare porous scaffolds, such as vacuum freeze drying [11], particle leaching [12,13], microsphere sintering [14], gas foaming [15,16,17], and electrostatic spinning [18,19], among others [20,21,22,23]. However, some of these strategies suffer from certain limitations in generating efficient porous scaffolds for engineering bone, such as the residue of the pore-forming agent, the difficulty in controlling the shape of the pore, and the complexity of the preparation process. To this end, 3D printing has emerged as a promising technology for fabricating geometrically defined porous architectures in 3D, thereby efficiently improving the physiological relevance of tissues and overcoming the significant limitations of various scaffold-based approaches, including the use of a pore-forming agent and restricted control over the 3D architectures, which reduced reproducibility [21,24,25]. In general, the process of 3D printing is accomplished as follows: first, the 3D model is built through computer-aided design (CAD); then, the 3D model is divided into two-dimensional (2D) models, and the 2D models are stacked together to accomplish the 3D structures. Then, the 3D structure is processed to get the desired architectures. Numerous polymeric inks are used for printing the 3D architectures of tissues and organs. In this vein, gelatin (Gel) [26], nano-hydroxyapatite (n-HA) [27,28], and poly(lactide-*co*-glycolide) (PLGA) [29,30] are the most preferred polymers in the 3D printing of biomimetic structures. However, they suffer from the disadvantages of poor mechanical properties, low cell adhesion rate, and poor hydrophilicity when used individually. 

Herein, we present a novel hybrid strategy based on 3D printing and freeze-drying techniques to fabricate biocompatible polymeric scaffolds based on Gel, n-HA, and PLGA for bone tissue engineering. Initially, the PLGA scaffolds were printed using the 3D printing technique, and then, the 3D PLGA scaffolds were subjected to a wetting process to enhance their biocompatibility and feasibility in depositing the mixture of n-HA and Gel over their surfaces. Further, we systematically investigated the physicochemical attributes and mechanical properties of the scaffolds through various physical characterization techniques, such as Fourier-transform infrared spectroscopy (FTIR) for determining the changes in the chemical functional groups, differential scanning calorimetry (DSC) and thermogravimetric analysis (TGA) for determining the weight loss events that indicate the thermal stability, and field emission scanning electron microscope (FE-SEM, S-4800, HITACHI, Tokyo, Japan) for exploring the morphological attributes. On the other hand, the contact angle test was carried out for clarifying the wetting of the PLGA scaffolds. Biodegradability, as well as biocompatibility attributes of the Gel/n-HA/PLGA scaffolds, were determined to explore their application. Initially, the cytocompatibility of various scaffolds were tested in three different cell lines: mouse fibroblast cells (L929), embryonic osteoblast precursor cells (MC3T3-E1), and rat bone marrow stromal cells (BMSCs). Further, the adhesion, as well as the growth studies, of osteoblasts were measured using embryonic osteoblast precursor cells (MC3T3-E1) alone, as these are also mostly used due to their significant advantages such as rapid differentiation. Moreover, the MC3T3-E1 cells are preosteoblasts that have been used extensively as an in vitro model system to examine osteogenic differentiation, revealing their suitability as an in vitro model for biomaterials testing. Finally, the effect of porous scaffolds on the differentiation of the MC3T3-E1 cells was shown by analyzing the osteoblast-specific markers, revealing the underlying implications of porous scaffolds on engineering bone tissue.

## 2. Materials and Methods

### 2.1. Materials

The PLGA copolymer was purchased from the Daigang Biological Co. Ltd. (Jinan, China). Potassium bromide (KBr), Gel, absolute ethanol (99.8% purity), dichloromethane (DCM, 99.8% purity), and glutaraldehyde (99.8% purity) were purchased from the Sinopharm Chemical Reagent Co., Ltd. (Shanghai, China). n-HA (70% of purity) was purchased from the Weijing nano Co., Ltd. (Huizhou, China). All other compounds were of analytical purity and were used without any further purification. Cell lysate, an alkaline phosphatase (ALP) assay kit, 3-(4,5-dimethylthiazol-2-yl)-2,5-diphenyltetrazolium bromide (MTT), the mouse osteocalcin (OC) enzyme-linked immunoassay (ELISA) kit, and the collagen type-I (COL-I) ELISA kit were purchased from Jiancheng Ltd. (Nanjing, China).

### 2.2. Fabrication of 3D PLGA Scaffolds

A commercial 3D printer (Regenovo 3D Bio-Architect, Hangzhou, China) was used to print the 3D microfibrous scaffolds based on the extrusion-based 3D printing process. Herein, the PLGA was added into the high-temperature cylinder of the 3D Bio-Architect, and the conditions provided for printing were adjusted at a printing temperature of 200 °C, printing pressure of 0.17 Mpa, and printing speed of 12 mm/s. Moreover, the specifications relevant to the gel dispenser were adjusted such that the width and length of the fibrous scaffold were 15 and 15 mm, respectively, at the altered thickness and filling spaces.

### 2.3. Hydrophilicity Assessment of Scaffolds

The hydrophilicity assessment of the PLGA scaffolds was tested by following the procedure below. Initially, the scaffolds were placed in the anhydrous ethanol for 6, 9, and 12 h. Next, the scaffolds were separated, and the remnants of the ethanol on the scaffold surface were wiped off. These scaffolds were dried naturally, and the scaffolds of each group were then placed in fresh tubes containing physiological saline for 0.5, 2, 24, and 48 h. The water on the surfaces of the scaffolds was removed by water absorbent paper, and the weight of the scaffolds and subsequently the water absorption rate was determined.

### 2.4. Preparation of the Gel/n-HA/PLGA Scaffolds

**Figure 1:**
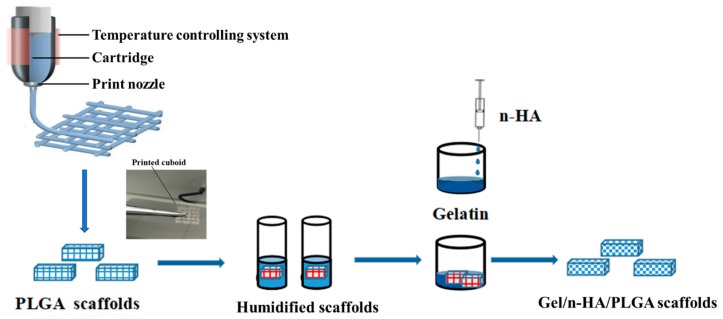
Schematic illustration representing the fabrication of the gelatin (Gel)/nano-hydroxyapatite (n-HA)/poly(lactide-*co*-glycolide) (PLGA) scaffolds. Initially, the PLGA microfibrous scaffolds were prepared by a three-dimensional (3D) printing method, and then, their humidified 3D scaffold architectures were dispersed in Gel and n-HA for the preparation of the Gel/n-HA/PLGA scaffolds.

Furthermore, we extended our study to fabricate the porous Gel/n-HA/PLGA scaffolds by immersing the humidified 3D PLGA scaffolds (shortly denoted as the humidified scaffolds in the later sections) in a Gel/n-HA solution (1:5 ratio, 4/20% in water). On the other hand, the effect of Gel concentration on the porosity was also tested by varying the amounts of Gel (2%, 3%, and 4%) at a constant ratio with n-HA. These scaffolds were further frozen and lyophilized for 24 h. The Gel was crosslinked with glutaraldehyde (0.25%), and the Gel/n-HA/PLGA scaffolds were obtained after drying under vacuum. The preparation process of the Gel/n-HA/PLGA scaffolds is shown in Figure 1.

### 2.5. Physical Characterizations

The functional groups were recorded by FTIR on a Bruker Alpha spectrometer at a specified wavenumber range (4000–500 cm^−1^) using the KBr pellet method. The samples were prepared by mixing them with KBr at a ratio of 1:200 and pressing them in a 7 mm die, and the spectra were then recorded. X-ray diffraction (XRD) data were recorded on an XRD spectrometer equipped with graphite monochromatized Cu–Kα radiation (λ = 0.15405 nm, 2-theta from 10 to 35 degrees). The thermal behavior of the scaffolds was determined by differential scanning calorimetry (DSC) analysis. The sample was placed in the alumina crucible and subjected to a temperature on a scale of 0–100 °C at 10 °C/min heating rate under argon as a carrier gas (20 mL/min). In addition, the thermogravimetric analysis was also performed by heating the sample in the range of 20–600 °C at a heating rate of 10 °C/min under argon as a carrier gas. The contact angle of the humidified scaffolds, as well as that of the 3D PLGA scaffolds, were measured by the contact angle admeasuring apparatus. The surface morphology of the scaffolds was elucidated using FE-SEM. The samples were prepared by adhering onto an aluminum stub with a thin self-adhesive carbon film and then coated with an ultrathin layer of gold, and the images were captured. Confocal laser scanning microscope (CLSM, TCS SP5, Leica, Germany) was used to capture the stained cells. The porosity of the composite scaffolds was measured by a gravity bottle method by immersing the scaffolds in anhydrous ethanol. 

### 2.6. In Vitro Cytotoxicity Assay

In this study, we used mouse fibroblast cells (L929 cell line), mouse embryonic osteoblast precursor cells (MC3T3-E1), and rat bone marrow stromal cells (BMSCs) for measuring the biocompatibility of our scaffolds. All the cell lines were purchased from the Shanghai Institute of Biochemistry and Cell Biology, China. The MC3T3-E1 and BMSCs were maintained in minimal essential medium-alpha (α-MEM; Gibco, Thermo Fisher Scientific, Waltham, MA, USA), and the L929 cells were maintained in Dulbecco’s modified Eagle’s medium (DMEM, Gibco), containing 10% (*v*/*v*) Fetal bovine serum (FBS) and 1% penicillin/streptomycin, at 37 °C in 5% CO_2_ atmosphere.

Cell cytotoxicity was measured using the WST assay. Before the treatment, all the scaffolds were sterilized by ultraviolet irradiation. The cells were seeded at a density of 5000 cells per well of a 96-well plate. After 24 h of incubation for proper cell attachment, the medium was replaced by the aliquots of medium exposed to the scaffolds at different concentrations (0.25, 0.5, 1.0, 2.0, and 5 mg/mL). A group containing the medium alone was set as the control. Furthermore, the reagent solution (10%, *v*/*v*) was added to each well and allowed to stand for 3 h. Then, the absorbance at λ = 450 nm was recorded, and the percentage of viable cells was calculated with reference to the absorbance of a control group of cells.

### 2.7. Biodegradation

The degradation experiments were carried out by maintaining the conditions that mimic the physiological environments concerning the pH and the temperature of the medium. The prepared scaffolds (the 3D PLGA scaffolds, as well as the Gel/n-HA/PLGA scaffolds) were exposed to the phosphate buffered saline (PBS) solution (pH-7.4) and placed in the rotatory incubator maintained at 37 °C at 200 rpm for predetermined time periods (1, 5, and 9 weeks). Finally, the scaffolds were separated, and the samples were prepared and subjected to SEM imaging.

### 2.8. Cell Adhesion and Viability Assays

The MC3T3-E1 cells were seeded at a density of 500,000 cells/mL/well on the 3D PLGA scaffolds, the humidified scaffolds, and the Gel/n-HA/PLGA scaffolds. After 4, 12, and 24 h of exposure, the medium was removed, the scaffolds were washed twice with PBS, and the non-adherent cells were then collected and counted using a hemocytometer. Furthermore, the following Equation (1) was used to calculate the cell adhesion rate:

Cell adhesion rate = 1 − (non-adherent cells/seeded cells).(1)

In addition to cell adhesion rate, we have also visualized the cells attached to the scaffold surface using SEM observations after 4, 12, and 24 h of exposure time periods. Finally, the viability of cells in different scaffolds was determined by assessing the live–dead cells at the end of the culture. The cells were stained with acridine orange and ethidium bromide (AO-EB) for 20 min at 37 °C and then washed twice with PBS. Images showing the stained cells were captured using the CLSM imaging technique after 4 h and 8 h of incubation with the cells.

### 2.9. Cell Proliferation Assay

The cell proliferation was detected using the MTT assay, which was reported previously [31]. The MC3T3-E1 cells were seeded at a density of 1 × 10^4^ cells per well and incubated overnight for proper cell attachment. The degradation solutions of the Gel/n-HA/PLGA scaffolds (50 μL) that were exposed to the cells was considered to be the experimental groups. MEM was used as a control group. They were further incubated for predetermined time intervals (2–10 days), and then, the MTT solution (1 mg/mL of MTT in PBS) was added and incubated for 4 more h. At the end of incubation, the medium was pipetted out, and the formazan crystals were dissolved by adding DMSO. Finally, the absorbance was measured at 570 nm using an ELISA reader.

### 2.10. ALP Activity and Total Protein Content

The cell lysate was taken, and the ALP content was determined according to the manufacturer’s instructions. The standard curve of the bovine serum albumin (BSA) standard was constructed according to the instruction of the BSA protein content detection kit, and the content of the cell secreting protein in the scaffolds was detected [23].

### 2.11. OC and COL-I Content

To determine the OC and COL-I content, the extracts (50 μL) of the 3D PLGA scaffolds, the humidified scaffolds, and the Gel/n-HA-PLGA scaffolds were added to 500 μL of MC3T3-E1 cell suspension (5 × 10^5^ cells/mL) in a 24-well plate. Various sterilized scaffolds were respectively placed in the complete culture base for 24 h, respectively, and then transferred to a 24-well plate. Then, 550 μL of MC3T3-E1 cell suspension (5 × 10^5^ cells/mL) was added to the scaffold in addition to a blank group containing 550 μL of MC3T3-E1 cell suspension (5 × 10^5^ cells/mL). Cell-specific metabolic activities were studied after 3, 5, and 7 days by measuring the OC and COL-I expression levels. The changes of OC were detected by the mouse OC ELISA kit (Jiancheng Ltd., Nanjing, China); COL-I expression was detected by the COL-I ELISA kit (Jiancheng Ltd., Nanjing, China).

### 2.12. Statistical Analysis

All results subjected to significance tests were presented as the mean ± standard deviation (*n* = 3). The statistical analysis of all the experimental data was performed using SPSS version 19.0. Analysis of variance (ANOVA) single factor analysis was conducted at a defined level of statistical significance of *p* < 0.05.

## 3. Results and Discussions

### 3.1. Physical Characterizations

**Figure 2:**
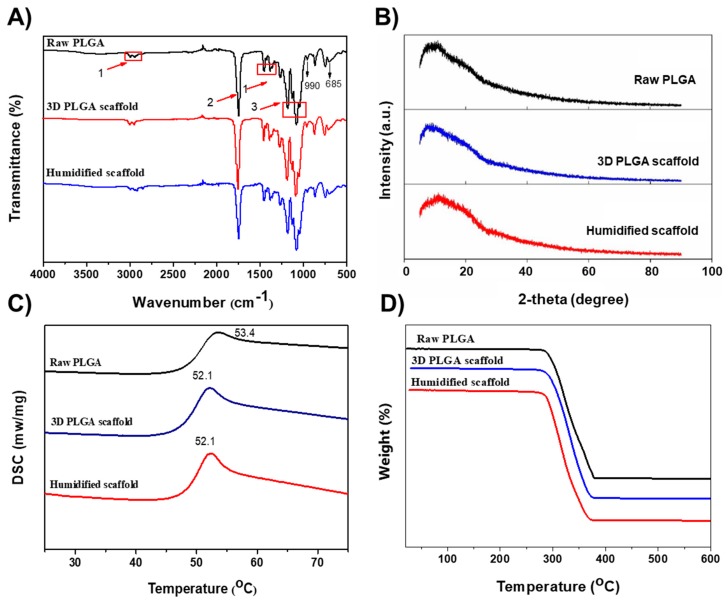
Physical characterization of the 3D PLGA scaffolds, as well as of the humidified scaffolds, in comparison to the raw PLGA. (**A**) FTIR, (**B**) XRD, (**C**) differential scanning calorimetry (DSC), and (**D**) TGA, of the raw PLGA, the 3D PLGA scaffolds, and their humidified scaffolds.

Various physical characterization techniques were utilized to illustrate the changes in the PLGA, if any, during the 3D printing and subsequent wetting process, and then, we compared the respective results of these scaffolds with the raw PLGA. Initially, the characteristic functional groups of the PLGA were identified by FTIR spectroscopy. As shown in Figure 2A, the characteristic peaks of the PLGA are divided into 3 portions as marked in the figure. “1” indicates the absorption peak at 2830 and 2920 cm^−1^ that was attributed to –C–H stretching vibrations. The sharp peak at around 1650 cm^−1^ (“2”) corresponds to the –C=O vibrations of the carboxylate group. A couple of peaks at around 1050–1300 cm^−1^ (“3”) were ascribed to ester group vibrations. In addition, the peaks at 990 and 685 cm^−1^ correspond to the C-H bending vibrations. The mentioned IR absorption peaks of the raw PLGA, the 3D PLGA scaffolds, and the humidified PLGA scaffolds were approximately the same, demonstrating that no significant changes in the functional groups were observed during the printing process.

The XRD patterns of the 3D PLGA scaffolds, along with the humidified scaffolds and the raw PLGA, are shown in Figure 2B. The spectra represented that there exist a halo-shaped characteristic peak, with no additional peaks in the 3D PLGA scaffolds similar to those of the raw PLGA, indicating that the materials were in the amorphous state. Furthermore, the thermal behavior of the PLGA scaffolds was measured by DSC analysis, (Figure 2C). The PLGA thermogram displayed a peak at 52 °C that corresponds to the glass transition temperature of the polymer, which can represent the changes in the structural rearrangement. The 3D PLGA scaffold, after high-temperature melting, has shown slight changes in the transition temperature (52.5 °C) compared with that of the raw PLGA (53.4 °C), revealing the structural arrangement of the PLGA leading to the significant heat transfer [32]. However, there was no significant influence on the structure of the 3D scaffold after ethanol treatment (i.e., the humidified scaffold). Further, the degradation behavior of the PLGA was demonstrated by TGA analysis. Figure 2D depicts that the scaffolds have shown weight loss at around 270–370 °C, revealing that the PLGA was degraded in that temperature range. However, the weight loss rates of all the samples were almost the same. It is evident from these results that the melting temperature during the printing of the scaffolds (200 °C) had no significant influence on the chemical structure and molecular rearrangement of the PLGA.

### 3.2. Contact Angle Test

**Figure 3:**
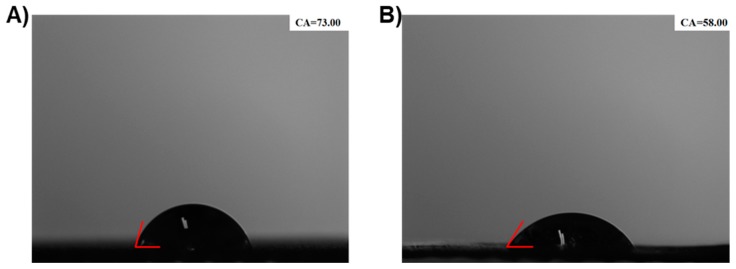
Contact angle test for determining the extent of the wetting of the 3D PLGA scaffolds. (**A**) 3D PLGA scaffolds, and (**B**) Humidified scaffolds.

To clarify the Gel-n-HA composite deposition, the wettability of the scaffolds was measured. The surface hydrophilicity of the 3D scaffolds was evaluated by the contact angle test. As shown in Figure 3, after the wetting treatment, the contact angle of the PLGA decreased from 73° to 58° demonstrating the critical improvement in the hydrophilic properties of the materials. Though the surface of this scaffold was not in contact with the cells, in some instances, the hydrophilic surface of the scaffold was more conducive for cell adhesion and proliferation and cytoskeleton formation after the distribution of the cells in the pores. On the other hand, the scaffolds with more moisture on their surfaces had significant enhancement of their cellular compatibility, which was attributable to the hydrophilic surface, as well as the porous architecture of the scaffold.

**Figure 4:**
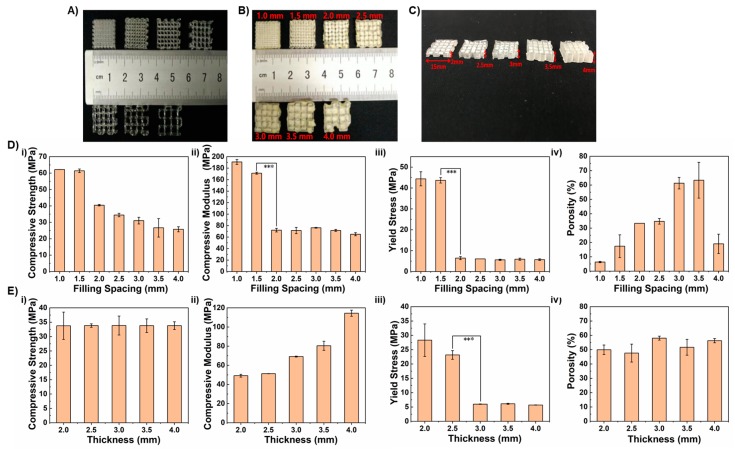
Optical images of various scaffolds and mechanical, as well as physical, properties of the Gel/n-HA/PLGA scaffolds. Optical Images showing the (**A**) printed 3D PLGA scaffolds and their successive Gel/n-HA/PLGA scaffolds at different (**B**) filling spaces and (**C**) thicknesses. (**D**) Graphical representation showing the changes in the mechanical and physical properties (**i**. compressive strength; **ii**. compressive modulus; **iii**. yield stress; and **iv**. porosity) of the Gel/n-HA/PLGA scaffolds at different (**D**) filling spaces and (**E**) thicknesses of the scaffold. *** represents *p* < 0.005.

As shown in Figure 4A, it is evident that the microfibrous 3D PLGA scaffolds, as well as their successive Gel/n-HA/PLGA scaffolds with different filling spacings and different thicknesses, were successfully fabricated by 3D printing and the deposition of the Gel/n-HA composite, respectively. The effect of the filling space, as well as the thickness of the printing layer on the mechanical properties, as well as porosity of the scaffolds was investigated. Herein, the filling space was changed by keeping the thickness (3 mm) constant. The scaffolds with measurements of 15 × 15 × 3 mm (*X*, *Y* and *Z* axis) were printed at altered filling spaces of 1, 1.5, 2, 2.5, 3, 3.5, and 4 mm (Figure 4B), as well as altered thicknesses of 2, 2.5, 3, 3.5, and 4 mm at 15 × 15 mm (*X* and *Y* axis) (Figure 4C), and the respective scaffolds were filled with Gel/n-HA yielding the Gel/n-HA/PLGA scaffolds. Furthermore, the mechanical properties, such as the compressive strength, compression modulus, and yield stress of the scaffolds were measured. As shown in Figure 4D, the filling distance mainly affects the composition of composite scaffolds, revealing that the smaller the filling space of the PLGA scaffold, the smaller the amount of Gel/n-HA composite solution was filled. Thus, there is a more significant proportion of the supporting PLGA in the scaffold, resulting in a relatively complete and dense structure with better mechanical properties. The results have shown that the compression strength, as well as the compression modulus of the Gel/n-HA/PLGA scaffolds, decreased gradually from 62.16 to 25.73 MPa, and 190.87 to 76.23 MPa, respectively, with the increase of the filling space. Notably, when the filling distance increased from 1.5 to 2 mm, the compression modulus decreased significantly. Moreover, the yield stress also decreased with the increase of the filling spacing, and the decrease of yield stress was very significant when the filling space was increased from 1.5 to 2 mm. Contrarily, the porosity of the scaffolds was increased from 6.36 to 63.33 % when the spacing between the supports increased from 1 mm to 3.5 mm, revealing that the amount of deposited Gel/n-HA inside the scaffold was low at 1 mm of spacing and *vice versa*. Further increase in the spacing of the scaffold to 4 mm resulted in an enormous drop in the porosity due to distorted shapes, leading to a reduction of fine pores. Furthermore, the effect of the thickness of the printed scaffolds on the mechanical properties, as well as porosity was investigated by printing the scaffolds with various thicknesses at a constant filling space of 3 mm. It is evident from the figure that the thickness of the Gel/n-HA/PLGA scaffolds had no effect on the compressive strength of the scaffolds. However, the compression modulus had shown gradual increment from 49.16 to 114.44 MPa with the increase of the thickness of the scaffold from 2 to 4 mm. Contrarily, the yield stress gradually decreased with the increase of thickness significantly from 2.5 to 3 mm. On the other hand, similar to the compressive strength, it was shown that there was no significant change in the porosity of the scaffolds when the filling space was constant. Hence, these mechanical properties, as well as the porosity determinations, demonstrated that these scaffolds deposited with composite inks of Gel/n-HA showed better mechanical properties and enormous porosity suitable for tissue engineering.

**Figure 5:**
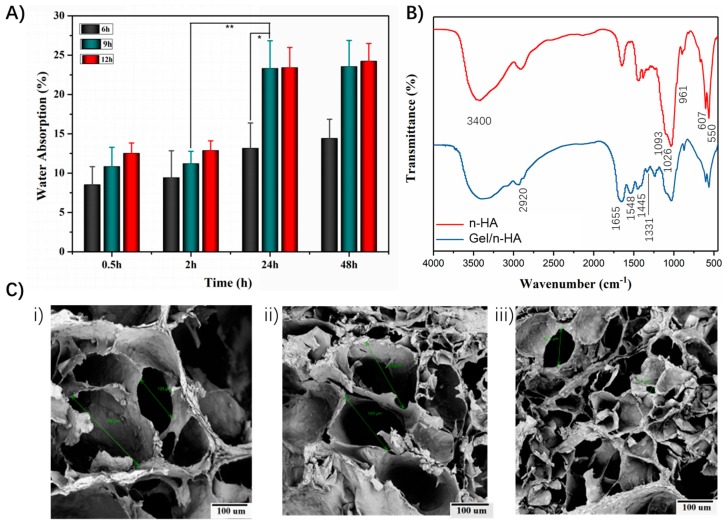
Physicochemical characterization of scaffolds after the deposition of Gel/n-HA. (**A**) Water absorption capacity of the 3D PLGA scaffolds at different exposure times (6, 9, and 12 h). (**B**) FTIR spectra of n-HA, and the Gel/n-HA composite. (**C**) SEM images of the Gel/n-HA/PLGA scaffolds at different amounts of Gel. (**i**: 2; **ii**: 3; **iii**: 4 g). * represents *p* < 0.05. ** represents *p* < 0.01.

More often, the protein expression, gene sequence, and the interactions between the cells and the extracellular matrix (ECM) with various biochemical cues, affect the behavior of the cells in the tissues and organs [33]. In this context, it is highly necessary to prepare the biomimetic scaffolds that resemble the ECM to facilitate the microenvironment for growth and efficient differentiation of cells. To address this issue, we subjected our 3D scaffolds to a humidification/wetting process, and furthermore, the polymeric composite ink of Gel and n-HA was deposited. As shown in Figure 5A, the water absorption efficacy of the scaffolds was increased from 13.2 to 23.3%, when the anhydrous ethanol treatment time of the PLGA scaffolds was increased from 6 to 9h. The reason behind the improvement of the hydrophilic property is that the treatment of the negatively-charged PLGA scaffolds with the anhydrous ethanol can adjust the proportion of scaffold’s surface charge, resulting in the reduction of negative charge [34]. However, after a further increase in the exposure time from 9 to 12 h, the water absorption rate of the scaffolds showed no significant change (i.e., from 23.3 to 23.4%, respectively). The saturation point might have been achieved by the scaffolds due to the long exposure time or might reached the saturation point of water absorption.

The successful formation of the Gel/n-HA complex was determined by the FTIR spectra recordings in comparison with the pure n-HA [35,36,37]. Figure 5B depicts the corresponding peaks of n-HA, as well as the Gel/n-HA composite. The broad absorption peak at around 3400 cm^−1^ was ascribed to the OH vibration absorption peak due to the hydroxyl groups or physisorbed water molecules. The spectrum of n-HA has shown the fundamental vibration peaks of phosphate at 550, 607, 961, 1026, and 1093 cm^−1^, confirming the n-HA structure. The sharp peaks at 2920 and 1655 cm^−1^ account for C-H stretching vibration and C=O stretching vibration of carboxylate, respectively. Moreover, a small peak at around 1445 cm^−1^ was inferred to be the characteristic absorption peak of the N-H stretch of tertiary amines. Additional peaks, such as a sharp peak at 1548 cm^−1^ for the N-H stretching vibration peak and the carbonate stretching vibration at 890–800 cm^−1^, correspond with the combination of Gel and n-HA. Most importantly, a peak at 1331 cm^−1^ was attributed to the Ca-COO^-^ vibration, confirming the formation of a coordination linkage between the calcium ions of HA and the carboxyl group of Gel [38]. These spectral confirmations provide evidence for the adequate formation of the Gel and n-HA complex. Figure 5C illustrates the surface morphologies of the Gel/n-HA/PLGA scaffolds at different amounts of Gel at a constant ratio of Gel and n-HA. It is evident that there was a significant difference in the porosity of the scaffolds with the increase in the concentration of Gel, indicating that the sizes of the pores were smaller and more uniform at higher amounts of Gel.

### 3.3. Biocompatibility Assay

**Figure 6:**
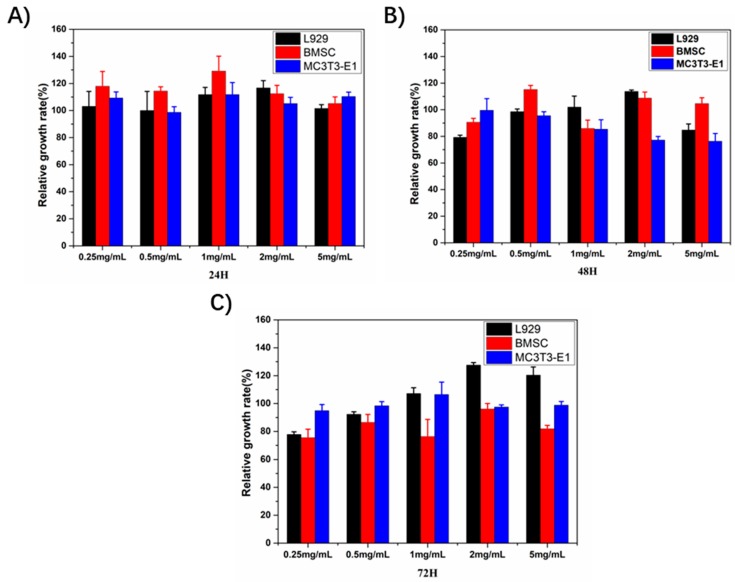
Biocompatibility measurement of the Gel/n-HA/PLGA scaffolds. The relative growth rate of the various cell lines (L929, BMSCs, and MC3T3-E1 cells) after exposure to the extract of the Gel/n-HA/PLGA scaffolds at different time intervals, (**A**) 24, (**B**) 48, and (**C**) 72 h.

The biocompatibility of the Gel/n-HA/PLGA scaffolds was performed using L929, MC3T3-E1, and BMSC cells at different exposure times of 24, 48, and 72 h using the WST assay, and the results are illustrated in Figure 6. The rationale behind choosing these cells is that they were often used for bone tissue engineering by depositing them in the 3D scaffolds. The polymers, Gel, n-HA, and PLGA, used in this study are generally considered to be biodegradable and biocompatible materials and are often utilized for various biomedical applications, including tissue engineering and drug delivery. Despite their compatibility, the polymers, when subjected to processing technologies during device preparations, in some instances show changes in their confirmations, yielding toxic substances. Thus, it is a prerequisite to measure biocompatibility attributes in that application area. Herein, the biocompatibility of the scaffolds was assessed in the aforementioned cells at different concentrations of the extract of the scaffolds in the medium for various exposure times. Indeed, it was observed that most of the cells at each concentration grew well, and the relative growth rate was over 90% (Figure 6A) in all the treatment groups at different exposure times (Figure 6B,C). Thus, the Gel/n-HA/PLGA scaffolds resulted in excellent biocompatibility.

### 3.4. Biodegradation

**Figure 7:**
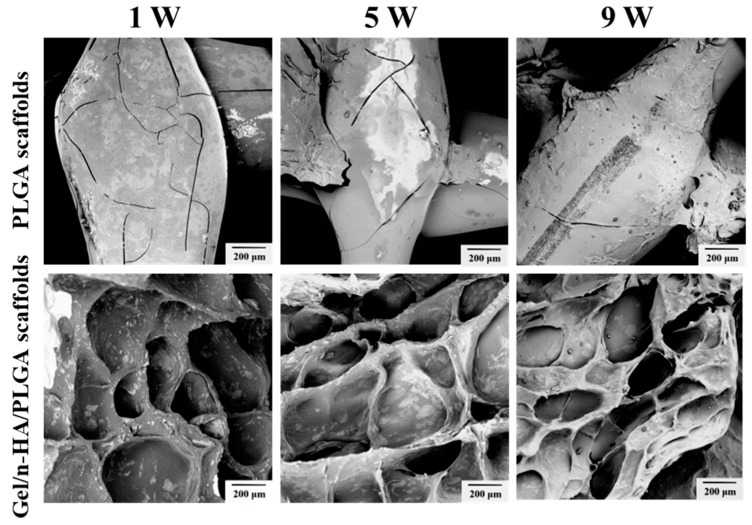
Biodegradation behavior of various scaffolds in physiological fluids. SEM images representing the biodegradation behavior of various scaffolds (the 3D PLGA scaffolds and the Gel/n-HA/PLGA scaffolds) exposed at different time intervals (1, 5, 9 weeks).

Biodegradation is an essential attribute to be considered while preparing the devices for biomedical applications. The initial understanding of the degradation process of scaffolds is necessary as it plays a crucial role in implanting them in the body. More often, the ideal scaffolds used in tissue engineering should have controllable degradation, and the rate of degradation must also be adapted to the speed of tissue regeneration. The biodegradation behavior of the 3D PLGA scaffolds, as well as the Gel/n-HA/PLGA scaffolds, were observed by exposing them to the buffers mimicking the physiological fluids for different exposure times (1, 5, and 9 weeks). Indeed, the results in Figure 7, demonstrated that the PLGA scaffolds showed a slightly brittle nature; there was a sign of degradation in the very first week, and remained in the subsequent weeks of exposure. However, there was not much degradation observed in the 9th week, which is attributable to the excellent mechanical properties. On the other hand, with respect to the composite polymers, Gel/n-HA deposited over the surface of the 3D PLGA scaffold showed an intact nature in the beginning, but the porous architectures dismantled after long-time exposure (9th week), indicating that the coating of polymers would create a loose surface and be prone to biodegradability, which is attributable to the porosity of the scaffolds.

### 3.5. Cell Adhesion Assay

**Figure 8:**
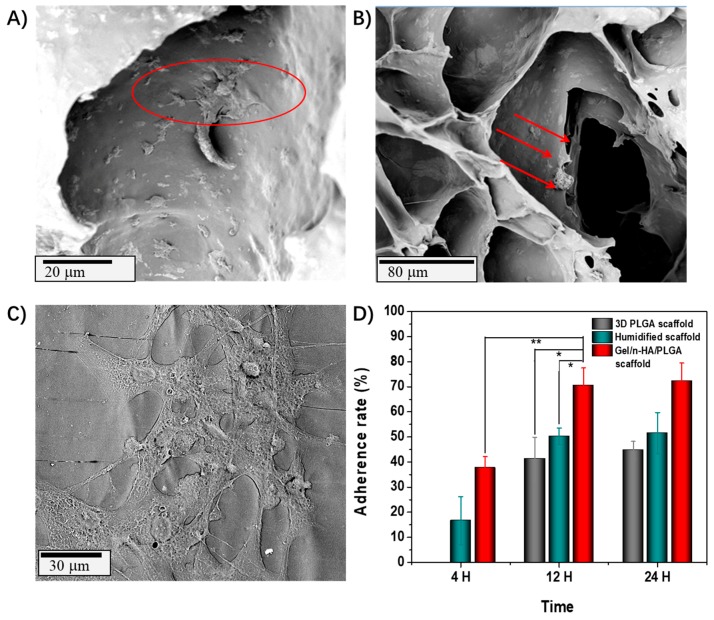
Cell adhesion rate of various scaffolds. SEM images showing the adhesion of embryonic osteoblast precursor cells (MC3T3-E1 cells) onto the Gel/n-HA/PLGA scaffolds exposed to various time intervals (**A**) 4 h, (**B**) 12 h, and (**C**) the magnified view of the image captured after 24 h of exposure. (**D**) Graphical representation showing the adherence rate of various scaffolds at different exposure time points. * represents *P* < 0.05. ** represents *p* < 0.01.

The first contact of the cells with the surface of the scaffold is an important attribute that determines the settling ability of the cells to conveniently arranging themselves onto the scaffold to facilitate the environment for their growth and differentiation [39]. In this context, the surface properties of the material play a significant role in affecting the cell adhesion process. Yang et al. [40] demonstrated that the adhesion between the cells and the material was activated by the interaction of the biomaterials and the proteins so that the adhesion rate on the material could indirectly reflect the cell differentiation. Herein, the cell adhesion rate of MC3T3-E1 on the scaffolds with different topographical morphologies was also analyzed (Figure 8). The cell adhesion study of the scaffolds was performed by exposing the scaffolds for different time intervals (4, 12, and 24 h), and the images were captured using SEM. The adhesion rate of the cells over the Gel/n-HA/PLGA scaffolds was significantly high, and they could explicitly arrange themselves in the porous, rough architectures of the scaffolds. As shown in Figure 8D, the MC3T3-E1 cell adhesion rate of the Gel/n-HA/PLGA scaffolds reached 70% after incubating the scaffolds for 24 h, which was significantly higher than that of the humidified scaffolds (51.52%) as well as the naked 3D PLGA scaffolds. It is evident that the moisture treatment altered the surface properties of the printed PLGA scaffolds. Furthermore, the addition of Gel/n-HA altered the surface area and roughness of the PLGA scaffolds, thereby improving the cell adhesion [41].

**Figure 9:**
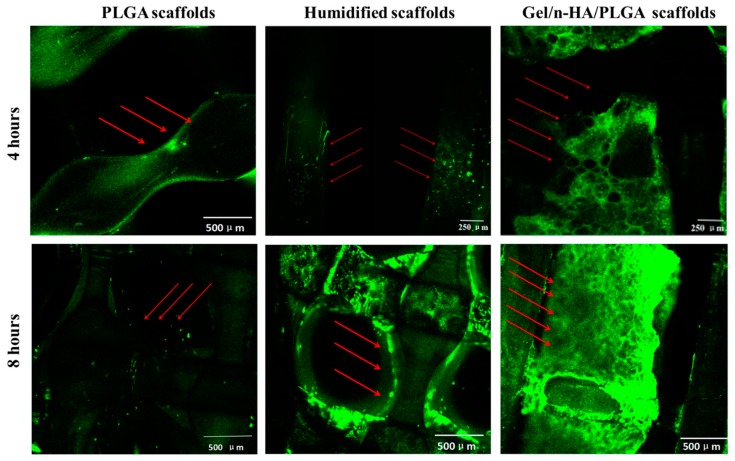
Cell viability analysis of various scaffolds. Confocal laser scanning microscope (CLSM) images of MC3T3-E1 cells showing that the cells were effectively deposited onto various Gel/n-HA/PLGA scaffolds after incubation for 4 and 8 h.

Based on the abovementioned results, we performed the distribution measurement of the viable cells on the Gel/n-HA/PLGA scaffolds based on the live–dead cell assay using the AO-EB stains for 20 min after 4 and 8 h of exposure time intervals. As shown in Figure 9, the results of the distribution of the cells on the Gel/n-HA/PLGA scaffolds demonstrated that the fluorescence in the scaffolds was significantly higher and intact compared with that in the 3D PLGA scaffolds. In addition, with the increase in time, the fluorescence in the Gel/n-HA/PLGA scaffolds increased, demonstrating that the exposure time showed significant influence on the arrangement and convenient differentiation of the cells. Moreover, the results were in agreement with the experimental results of the adhesion rate.

### 3.6. Effect of Degradation Products on MC3T3-E1 Differentiation

**Figure 10:**
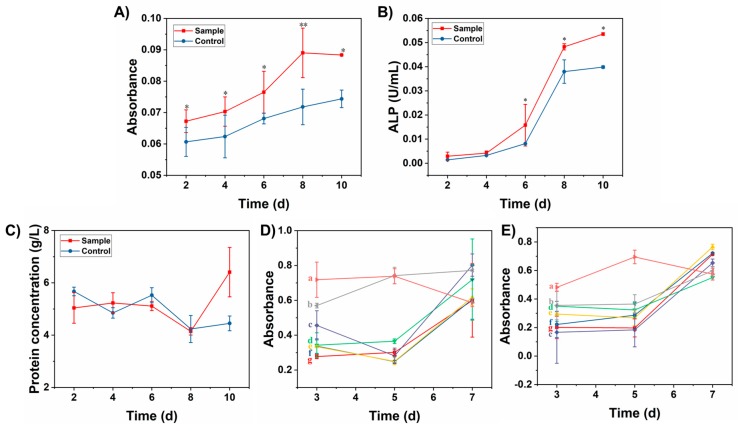
Effect of the degradation products on the osteoblast differentiation. Graphical representation showing the (**A**) proliferation rate of rat osteoblasts, (**B**) alkaline phosphatase (ALP) activity, as well as (**C**) Total protein synthesis, in the extract of the degradation solutions of the Gel/n-HA/PLGA scaffolds that have been incubated for predetermined time intervals. (**D**) osteocalcin (OC) and (**E**) collagen type 1 COL-I, levels were measured by exposing the cells to both extracts, as well as scaffolds, (a) blank group, and leaching solutions, as well as the stents of the samples of the 3D PLGA scaffolds (b, e), the humidified scaffold (c, f), the Gel/n-HA/PLGA scaffold (d, g), respectively. * represents *p* < 0.05. ** represents *p* < 0.01.

As shown in Figure 10A, the degradation solutions of the Gel/n-HA/PLGA scaffolds were co-cultured with the MC3T3-E1 cells as an experimental group, in addition to the control group of cells that were added with MEM alone. The MC3T3-E1 cells of both the groups have shown a considerable extent of proliferation in 6–8 days. Notably, in the process of co-culture for 10 days, the cell proliferation rate of the experimental group was significantly higher compared with that of the control group.

Furthermore, the osteogenic biomarkers such as ALP, total protein, OC, and COL-I were measured for exploring the efficiency of these Gel/n-HA/PLGA scaffolds toward osteogenic differentiation. Though ALP is widely distributed in bone, the kidneys, the liver, the intestines, and the placenta, it is one of the most common indicators to evaluate the secretory function of osteoblasts. In this context, the expression of ALP indirectly reflects the functional state, as well as the degree of differentiation of osteoblasts. Moreover, the ALP plays a significant role in bone calcification [42], which is also one of the primary indicators for evaluating the functions, as well as the early differentiation and maturation of mouse osteoblasts. Figure 10B depicts the results of the ALP activity of the scaffolds during the 10-day culture period. The highest level of ALP activity was recorded on the 10th day from cells cultured on the Gel/n-HA/PLGA scaffolds in comparison to that of the control group. This is evidence that the Gel/n-HA/PLGA scaffolds have shown an increased level of ALP activity. On the other hand, it was also observed that the total protein content (Figure 10C) was significantly increased during the 10-day culture period. The highest level of protein content was recorded on the 10th day in cells cultured on the Gel/n-HA/PLGA scaffolds in comparison with that of the control group, demonstrating that these scaffolds increased the protein content during the stages of differentiation, which is useful in monitoring the osteoblastic phenotype of the MC3T3-E1 cells.

Furthermore, the osteogenic potential of the MC3T3-E1 cells was assessed via the quantification of OC and COL-I expression of the cells incubated with the degradation solution, as well as the scaffolds with the control group treatment. From Figure 10D, it was found that the amount of OC increased gradually with the increase of incubation time. The experimental groups have shown higher OC content than that of the blank group. In the liquid group, the extract of the wet scaffold was higher than that of the ordinary scaffold extract group, and the Gel/n-HA/PLGA scaffolds could promote the secretion of OC in the MC3T3-E1 cells. COL-I is a specific collagen protein secreted by osteoblasts. It is the main bone structural protein, of which the bone matrix is mainly composed. The expression intensity of COL-I in the osteoblasts can reflect the functional state of the osteoblasts. In this experiment, the secretion of COL-I in the cells of the blank group and the experimental group was detected. From Figure 10E, it was found that the amount of COL-I produced by the MC3T3-E1 cells increased gradually with the increase of culture time. The levels of these osteoblast markers in the experimental groups were higher than those of the blank group. Notably, the secretions from the Gel/n-HA/PLGA scaffolds group were higher than the humidified scaffolds group, and those from the humidified scaffolds leaching solution group were higher than those from the 3D PLGA scaffolds group. The scaffolds prepared by the experiment had significantly promoted the secretion of COL-I in the MC3T3-E1 cells.

## 4. Conclusions

This study investigated the potential of porous Gel/n-HA/PLGA scaffolds suitable for use in bone tissue engineering. The Gel/n-HA/PLGA scaffolds prepared in this experiment had overcome the shortcomings of single material scaffolds. Moreover, the Gel/n-HA/PLGA scaffolds have exhibited good hydrophilicity, biocompatibility, and osteogenic characteristics and have shown no toxic substances during the degradation process. The degradation solution of the scaffolds could significantly promote the proliferation of osteoblasts, which was confirmed by the synthesis of ALP and total protein. Furthermore, the Gel/n-HA/PLGA scaffolds could also promote the proliferation of osteoblasts, which was confirmed by the synthesis of OC and COL-I. The 3D printing technology used in this experiment can conveniently produce porous scaffolds on demand, without the use of organic solvents. This innovative hybrid approach for generating the Gel/n-HA/PLGA scaffolds with great benefits will undoubtedly reveal the potential for new methods of treating of bone defects.
